# Architects of the Developing Brain: Cytoskeleton-Organizing Molecules in Neurodevelopmental Disorders

**DOI:** 10.3390/cells15060537

**Published:** 2026-03-17

**Authors:** Ksenia A. Achkasova, Pavel V. Subbotin, Vadim V. Zhukov, Anastasia E. Filat’eva, Victor S. Tarabykin, Elena V. Kondakova

**Affiliations:** 1 Institute of Neuroscience, Lobachevsky State University of Nizhny Novgorod, 23 Gagarin Ave., 603022 Nizhny Novgorod, Russia; achkasova.k@bk.ru (K.A.A.); pavel7156162@gmail.com (P.V.S.); wildnatural@mail.ru (V.V.Z.); filja.a@mail.ru (A.E.F.); elen_kondakova@list.ru (E.V.K.); 2 Department of Genetics and Life Sciences, Sirius University of Science and Technology, 1 Olympic Ave., 354340 Sochi, Russia

**Keywords:** cytoskeleton, corticogenesis, neurodevelopmental disorders, microtubules, actin filaments, intermediate filaments, microcephaly, lissencephaly, corpus callosum agenesis, synaptopathies

## Abstract

Different components of the cytoskeleton are very important determinants of brain development. They orchestrate multiple cellular processes involved in all phases of cerebral cortex development. In this review, we summarize current knowledge on the components of the cytoskeleton—microtubules, actin filaments, and intermediate filaments—and their roles in cortical development. We provide a detailed analysis of how cytoskeleton molecules control neuronal progenitor proliferation, neuronal migration, polarization, axon and dendrite specification and outgrowth, and synaptogenesis. We further examine how pathogenic variants in genes encoding cytoskeletal proteins or their regulators disrupt particular steps of neurogenesis and contribute to major neurodevelopmental disorders (NDDs). Focusing on NDDs such as microcephaly, lissencephaly, corpus callosum agenesis, and synaptopathies, we discuss consequences of cytoskeletal dysfunctions causing altered cellular behavior and clinical phenotypes. By linking molecular defects to developmental and phenotypic consequences, this review highlights the cytoskeleton as a central element in neurodevelopmental pathologies and underscores its potential as a target for future therapeutic strategies.

## 1. Introduction

The development of the cerebral cortex is a very complex multi-step process that plays a key role in human cognition, including thinking, memory, speech and consciousness. This process begins during embryogenesis with the proliferation of neural progenitors, followed by their differentiation, the migration of neurons to their target regions, as well as the growth of axons and dendrites and the formation of synaptic connections [1].

Throughout each of these developmental stages, the neural cell undergoes a series of changes that affect both its shape and structure, as well as its functional state. These processes are driven by the precise coordination of the cellular internal structure and its interactions with external signals, which are facilitated by the dynamic activity of the cytoskeletal elements. All components of the cytoskeleton, including microtubules, actin filaments, and intermediate filaments, play a significant role in neurogenesis [2].

Mistakes at any stage of neurogenesis lead to long-lasting structural and functional abnormalities that underlie the pathogenesis of neurodevelopmental disorders (NDDs) [3]. NDDs are a heterogeneous group of pathologies characterized by impaired cognitive, motor, and behavioral functions. These pathologies include autism spectrum disorders, intellectual disabilities, various cortical malformations, epilepsy, and others [4]. These conditions have a significant impact on the quality of life for millions of people worldwide and pose a significant challenge to the healthcare system.

At the molecular and cellular level, many of these abnormalities are associated with cytoskeleton dysfunction. The cytoskeleton in neurons not only serves a structural and support function, determining cell shape and mechanical strength, but also provides a substrate for intracellular transport, response to external signals, and the formation of neuronal circuits. Thus, microtubules serve as a base component for migration initiation and execution. They provide a scaffold for all proliferation steps, including the assembly of the mitotic spindle and establishment of its axis. On the other hand, they serve as transport routes for the transfer of organelles and other molecular cargo [2].

Actin filaments, in turn, are involved in the growth cone formation, axon navigation, as well as in the dynamics of dendritic spines and synaptic plasticity. Intermediate filaments perform a predominantly supportive function, maintaining the shape of the soma and neurites, and are involved in synaptic plasticity [5].

In recent years, a growing body of evidence suggests that pathogenic variants in genes encoding cytoskeletal components or their regulators are associated with diverse forms of NDDs. For example, pathogenic variants in genes encoding proteins in the tubulin family lead to a spectrum of pathologies manifested by impaired migration, disorganization of cortical layering, and impaired synaptic contact formation [6].

Similarly, defects in cytoskeletal regulators, such as microtubule-associated proteins (MAPs) or components of Rho GTPase-dependent pathways, cause impairments in axonal growth, dendritic spine stability, and synaptic plasticity [7].

One of the key features of the regulation of the various components of the cytoskeleton is its dynamics, coordination, and reactivity to both intracellular and extracellular signals. For example, the direction of axon growth is controlled by the coordinated interaction of microtubules and actin filaments inside the growth cone as well as by external signals.

During neuronal migration, the flexibility of the cytoskeleton plays a crucial role in enabling cell shape changes, organelle transport, and cell movement to reach target positions. These mechanisms are not only essential for maintaining the general architecture of the cortex but also contribute to the development of NDDs when they are disrupted [2].

Thus, understanding the role of cytoskeleton-organizing molecules in the occurrence of NDDs is of both fundamental and translational importance. On one hand, identification of specific defects in cytoskeleton regulation will allow for the identification of previously unknown pathogenic mechanisms, which may lead to the development of molecularly targeted therapeutic strategies to compensate for cytoskeleton disorders during early stages of development. On the other hand, linking the pathogenic variants of genes encoding cytoskeleton components to certain NDDs can be used to improve existing diagnostic panels aimed at the early detection of neurodevelopmental defects [3].

In this review, we summarize the current understanding of the molecules that organize the cytoskeleton, their dynamics, and the mechanisms of regulation in the context of cortical development. We focus on all the main components of the cytoskeleton and their role in the proliferation of neural progenitors, neuronal migration and polarization, axon and dendrite formation, and the establishment of synaptic connections.

We do not consider the cytoskeleton as just a collection of individual structural elements, but as a well-organized system that ensures the proper functioning of neurogenesis and neuronal differentiation. We discuss how pathogenic variants of genes encoding cytoskeleton components or their regulators lead to specific CNS defects. We emphasize the causal relationship between the dysfunction of a particular cytoskeleton component, the disruption of a specific stage of neurogenesis, and the development of the clinical phenotype of NDDs. Thus, this article aims to integrate experimental and clinical data, highlighting the cytoskeleton as a central player in the pathogenesis of multiple NDDs.

## 2. Molecular Components of Cytoskeleton

The cell cytoskeleton encircles the cytoplasm and internal compartments of the cell, facilitating almost all cellular events. The cytoskeleton consists of three macromolecular structures: microtubules (MTs), actin filaments (AFs), and intermediate filaments (IFs). This section provides basic information about these elements of the cytoskeleton, including their structure, properties, regulation, and functions.

### 2.1. Microtubules (MTs)

#### 2.1.1. Structure and Properties

MTs are a component of the cell’s cytoskeleton whose main functions are intracellular transport, the mitotic spindle organization and the maintenance of cell shape. As one of the three components of the cell’s cytoskeleton, they are made up by cylindrical polymers with a filamentous structure, formed by two isoforms of tubulin (α and β). The protein heterodimers are connected head-to-tail, forming a polarized linear protofilament with plus and minus terminus [8,9,10].

When 13 such protofilaments are bound together, an MT with a diameter of 25 nm and a length ranging from 1 μm to 100 μm is formed. The surface of MT has a pronounced negative charge due to the C-terminal regions, which are rich in glutamate [8,9,11].

MTs are closely associated with the microtubule organizing center (MTOC), where they are anchored at their minus ends [12,13]. The primary MTOC in eukaryotic cells is a set of two centrioles (centrosome), surrounded by the pericentriolar matrix [13].

The structural component of MT is tubulin molecules, one of the most abundant classes of proteins among eukaryotes, which belong to the superfamily of GTPases that assemble linear polymers [9,14]. The most studied are the cytoplasmic tubulins α and β, which form heterodimers that make up the bodies of MTs [15].

Both isoforms of tubulin can bind GTP. However, GTP bound to α-tubulin is not hydrolyzed during polymerization and cannot be exchanged, while GTP bound to β-tubulin is located on the surface of the dimer and can be hydrolyzed; however, after hydrolysis during polymerization, it is bound on the protofilament as GDP [16].

In addition, the tubulin family includes γ-tubulin, which is responsible for inducing the growth of new MTs and can also bind GTP in the γ-TuRC (γ-tubulin ring complex) [17,18]. A more important function of γ-tubulin is that, in cooperation with GRIP proteins (γ-tubulin ring proteins), it forms the core of MTs assembly [19,20]. The core serves as the basis for the subsequent growth of protofilaments composed of α and β tubulin [17,19].

#### 2.1.2. Microtubule Catastrophe and Rescue

One of the main remarkable features of MTs is their dynamics. MTs are capable of self-assembly and disassembly and dynamically change their organization depending on the needs of the cell [21]. At the same time, MTs are capable of stochastically switching between two states—polymerization and disassembly. Their dynamics are determined by the specific molecular properties of tubulin [22,23,24].

Each of the microtubule protofilaments is polarized, resulting in the microtubule being a polarized structure. The polarity of MT is due to the presence of a fast-growing plus end, which has an terminal β-tubulin molecule, and a slow-growing minus end, which has terminal α-tubulin [25]. MTs are assembled and disassembled by adding and releasing monomers from the ends, respectively.

The growth and disassembly of MT are mediated by two polymer states: rescue, in which the microtubule grows, and catastrophe, in which the microtubule shortens. The GTP–tubulins at the tip of the microtubule form a cap that stabilizes the growth of the microtubule. The loss or destruction of the cap will induce catastrophe. However, after the induction of catastrophe, the microtubule may suddenly stop shrinking and return to the growth phase, known as rescue [21,26,27,28].

#### 2.1.3. Microtubule Dynamics

MT dynamics depend on the hydrolysis of GTP and groups of proteins associated with MT. The assembly of MT is a spontaneous process, that is not mediated by GTP hydrolysis [25]. In contrast, it is disassembly that requires energy and depends on GTP hydrolysis. It was demonstrated in experiments when GTP molecules were replaced by GTP GMPCPP (guanidyl 5′-α,β-methylenediphosphonate), a hydrolysis-resistant GTP analog. In such experiments, MTs were growing, but could not be disassembled [29].

#### 2.1.4. Microtubule Regulation

MTs are very dynamic structures. Their dynamics depend on many factors, such as the following: interaction with MAP, external stabilizing and destabilizing factors (e.g., chemical alkaloids), terminus regulators, and post-translational modifications of tubulin.

Thus, the destruction of MTs can be induced by temperature fluctuation, ionic balance in the cytoplasm, the pH, etc. At the same time, they have a very high mechanical resistance [30]. Also, MTs dynamics depend on some small molecules such as taxol or colchicine. Taxol, that is capable of passing inside MTs through small holes in the molecule, accelerates the assembly of MTs and stabilization. Colchicine and vincristine, on the contrary, are capable of destabilizing and destroying MTs [15,31,32].

MAPs actively regulate the growth and assembly of MTs and enhance their stabilization [33,34]. In particular, MAP2, MAP4 and tau-protein accelerate the polymerization of MTs, as well as stabilize MTs, preventing catastrophes [35,36]. Tau is one of the key neuronal MAPs, affecting the stability of axonal MTs, organelle transport and synaptic plasticity [37].

Post-translational modifications of tubulin play an important role in the regulation of MTs. Various modifications contribute to the neuronal polarization and interaction of MTs with motor proteins. A large pool of enzymes interacts with tubulin in order to carry out such modifications as tyrosination, de-tyrosination, acetylation, polyglycylation, polyglutamylation, palmitoylation, phosphorylation, and Delta2 modification [38,39,40,41,42].

MTs end-binding proteins also have a significant impact on the growth and development of MTs, regulating their dynamics. The EB family of proteins (EB1, EB2, EB3) interacts with the plus end of MTs and coordinates their growth. CLASP and CLIP170 proteins stabilize the plus end.

XMAP215 enhances polymerization. Among the minus end-binding proteins, CAMSAP/Patronin protects the end from depolymerization [43], and the protein ninein organizes MTs in the cell’s centrosome and stabilizes MTs in neuronal axons. However, the stabilizing function was discovered relatively recently: the loss of functional activity of ninein made MTs more sensitive to nocodazole, an agent that destabilizes MTs [43,44,45,46].

#### 2.1.5. Functions of Microtubules

MTs, as one of the three main components of the cytoskeleton, are involved in a wide range of cell functions. One of the most important functions of MTs is the formation of the mitotic spindle apparatus, which ensures the separation of chromosomes during mitosis. The mitotic spindle is assembled by subpopulations of dynamic MTs, motor proteins and MAP.

In this case, MTs provide the alignment of chromosomes along the metaphase plate and the subsequent segregation of sister chromatids to the poles of the spindle apparatus [47,48]. Kinetochore proteins (Ndc80, KNL1, CLASP1, CENP-E) bind to the lateral surface of MTs, and motor proteins, by binding to MTs, ensure the movement of chromosomes and determine their position in the cell [48].

It is also important that MTs form MTOC, centrosomes, and polar poles bodies. As microtubule nucleation sites, these structures determine the architecture of the cytoskeleton, position the cell nucleus, form the bipolar spindle structure, and ensure the separation of chromosomes [12,49,50]. The γ-TuRC complex, which binds to the pericentriolar material and the centriolar lumen, controls the formation of MT by binding to adapter proteins (NEDD1 and CDK5RAP2) [51].

The formation of cell–cell contacts is also mediated by MTs: MTs regulate the positioning of cadherins (N-cadherin) and ensure their proper functioning [52]. Another process that depends on MTs and motor proteins is protein cargo transport from the ER through the Golgi apparatus to target cellular compartments [53].

MTs interaction with motor proteins, such as kinesin and dynein, is required for the movement of organelles and macromolecular complexes towards the plus end and towards the minus end, respectively. This is particularly important for axonal retrograde and anterograde transport [54,55,56,57].

MTs span almost the entire free space of the cell, which determines the shape of the cell. This also applies to the neurites, such as axons and dendrites. Through their interaction with other elements of the cytoskeleton, such as actin through spectraplakin, the flexibility and rigidity of cells are regulated [58].

### 2.2. Actin Filaments (AFs)

#### 2.2.1. Structure and Properties

AFs consist of a highly conserved protein that exists in the form of G-actin monomers and AFs polymers-F-actin. Similarly to MTs, AFs are polar structures, with a rapidly growing plus end and slowly growing minus end.

The assembly of AFs occurs through polymerization, where actin monomers interact with bound ATP to form a filament. Once polymerized, actin hydrolyzes ATP into ADP and releases phosphate. ADP–actin-bound filaments undergo disassembly for subsequent recycling of the subunits [59,60,61]. The stability and mechanical flexibility of these structures are maintained through the presence of side ligands and magnesium and calcium ions, which contribute to their mechanical function in neurons, such as the formation of neurites, mechanical support, etc. [62,63].

#### 2.2.2. Assembly and Disassembly of the Actin Filaments

The key stages of the formation of the actin cytoskeleton are the processes of nucleation and elongation. Nucleation consists of two steps: the formation of an actin dimer and the subsequent formation of a trimer. The formation of dimers and trimers is slow; they quickly dissociate and are found in a cell in small quantities, due to which these stages are very unstable [64,65,66].

In fact, a stable oligomer is assembled after the addition of the fourth subunit. Subsequently, the elongation stage follows—the growth of the filament from two ends. At the same time, there are two key components that determine growth and shortening. First component: After the formation of the actin–ATP complex, the hydrolysis of ATP to ADP occurs, while the already attached sections become less stable, which promotes dissociation, especially at the minus end [66].

The second component: It involves the regulation of the growth rate through interaction with various two- and one-valent cations (magnesium and calcium) and other regulatory molecules (profilin, β-timosin, cofilin, formin) [62,67].

There are several pathways for the assembly of F-actin: the Arp2/3 complex, the Ena/VASP complex, and the formin complex. Their combined action forms the majority of the polymers, and the complexes complement each other: the Arp2/3 complex forms branched AFs, while the Ena/VASP complex forms long, unbranched filaments [68,69,70]. In endocytic and phagocytic cell structures, adhesive contacts, and lamellipodia, Arp2/3 assembles branched filaments under the control of nucleation-promoting factors [71].

Ena/VASP is localized at the distal end of lamellipodia, where it regulates the density and length of the branches generated by Arp2/3, and incorporates G-actin into the growing barbed ends [72]. Ena/VASP is also localized at focal adhesions and filopodia, contributing to the formation of unbranched F-actin [73,74]. Formins facilitate the assembly of F-actin within filopodia and stress fibers, and are also involved in vesicular transport, cytokinesis, and phagocytosis [75].

The ADF/cofilin proteins play a significant role in regulating the dynamics and properties of AFs. This complex is particularly important in neuronal cells. ADF, cofilin-1, and cofilin-2 bind both G-actin and F-actin. The cofilin family mediates the twisting and breaking of contacts, facilitating the disassembly and recycling of monomers, which highlights their role as a filament dynamics switch [76,77].

#### 2.2.3. Regulation of the Actin Filaments

One of the most important elements of cytoskeleton control is the regulation of their components by signaling through regulatory signaling pathways. For example, the Rho family of GTPases, including RhoA, RhoB, RhoC, Rnd3/RhoE, Rac1, and Cdc42, are central regulators of actin [78]. These proteins have GTPase activity and can bind to GTP and GDP, inducing the phosphorylation of cytoskeletal proteins.

When bound to GTP, they interact with target proteins, activating them. Their activity is regulated by interactions with guanine nucleotide exchange factors, which induce the release of GDP. GTPase-activating proteins (GAPs) act as inhibitors, catalyzing the hydrolysis of GTP. These GTPases promote the polymerization of actin in specific regions of the plasma membrane and compartments, leading to membrane protrusion or the regulation of vesicle movement. In particular, this process is involved in exocytosis and endocytosis, as well as the transport of proteins from the Golgi apparatus to the ER [78,79,80,81,82]. The GTPases RhoA, Rac1, and Cdc42 are responsible for the formation of lamellipodia and filopodia [83]. Additionally, the phosphorylation of GTPases regulates cell division, cell polarity, morphology, and other cellular processes [83,84].

Among the regulatory molecules that affect the actin cytoskeleton, one should also mention the Wiskott–Aldrich syndrome protein (WASP) and the verprolin-homologous protein family (WAVE). These proteins regulate the actin cytoskeleton through a different strategy, inducing actin polymerization through the Arp2/3 complex described above. This leads to the formation of a new actin filament that branches off from the previous one, promoting actin reorganization [85].

The depolymerization of AF, on the other hand, is influenced by cofilin, which is regulated by the LIM kinase (LIMK) [86]. LIMK directly phosphorylates cofilin, inactivating it and leading to an increase in the amount of polymerized actin [87].

#### 2.2.4. Functions of the Actin Filaments

The actin component of the cytoskeleton in the cell is the main mechanism for generating a driving force. Its work creates pushing (protruding) forces due to the polymerization of AFs, and pulling (contractile) forces due to the sliding of AFs along myosin filaments [88]. Structures that provide cell mobility and directional response—lamellipodia and filopodia—are predominantly formed due to the work of the actin component of the cytoskeleton [88,89].

Branched actin networks form a quasi-two-dimensional layer inside lamellipodia and push the cell membrane by polymerizing in the opposite direction. Filopodia comprise aligned bundles of actin, resulting in a finger-like structure [63]. These membrane projections are essentially actin networks. They determine filopodia movement due to the force generated by the polymerization of actin. The precise mechanism of the occurrence of the driving force due to the polymerization of actin is not completely understood [89], while it is well-established that the regulation of movement occurs due to the activity of Arp2/3 and Rac GTPases [67,90].

The maintenance of the cell shape and its change is also achieved due to the work of the actin component of the cytoskeleton—a thin layer of actin (cellular cortex) covers the plasma membrane from the inside on the back and sides of the cell. The rest of the cell is predominantly lined with a three-dimensional network of AFs, which connects the cytoskeleton to the extracellular matrix through focal adhesion sites [63].

### 2.3. Intermediate Filaments (IFs)

#### 2.3.1. Structure and Properties

IFs are the third component of the cytoskeleton, along with MTs and AFs. They are named due to their size compared to the other elements of the cytoskeleton: their diameter is 10 nm, ranging between the sizes of MTs and AFs [91,92]. Unlike MTs and AFs, which are polymers of a single protein, this component of the cytoskeleton consists of a number of fibrillar proteins, encoded by ~65 genes [93,94]. These proteins have a distinctly organized, elongated α-helical conformation, prone to the formation of double-stranded coiled spirals [91]. At the same time, unlike MTs and AFs, IFs are not polar structures.

All IFs have three components in their structure: a central alpha-helical rod domain and variable N-terminal and C-terminal tail domains. There are six main classes of intermediate filament proteins: class I (acidic keratins), class II (basic keratins), class III (vimentin, desmin, peripherin), glial fibrillary acidic protein (GFAP), class IV (NF-L or light neurofilament, NF-M or medium neurofilament, NF-H or heavy neurofilament, and α-internexin), class V (nuclear lamins), and class VI (nestin) [91,95].

It should be noted that, depending on the cell types, there are differences in the classes of proteins that make up IFs. For example, neurofilaments (IFs found in neurons) are characterized by a predominance of class IV proteins that form light, medium, and heavy subunits [96]. In contrast, developing neurons are dominated by class III proteins, particularly vimentin [97].

#### 2.3.2. Intermediate Filament Assembly and Dynamics

Intermediate filament proteins are self-assembling components, and IF assembly is not an energy-dependent process. The assembly of this cytoskeleton component occurs in several stages. In the first stage, protein aggregation occurs; subsequently, this aggregate elongates, forming a rod. Thus, the initiation or nucleation of filaments occurs [91]. Furthermore, the rod domains of two monomers line up in parallel, forming a dimer. Two dimers associate antiparallel, and a nonpolar tetramer is formed [98]. Next, eight tetramers are assembled laterally into unit length filaments, which are then annelated end-to-end, forming a nonpolar filament. The next stage leads to the emergence of a mature cytoskeleton component in the course of radial compactization with a diameter of 10 nm [98,99].

#### 2.3.3. Regulation of Intermediate Filaments

IFs are elements of the cytoskeleton that are involved in many signaling pathways and regulatory cascades. They serve as signal transducers initiated by kinases (PKC, MAPK, ERK1/2), phosphatases, and adapter proteins. This allows for the regulation of molecular signaling pathways and cascades, which modulates the mechanobiochemical signals that regulate the cell’s response [100,101]. The RhoA/ROCK pathway is primarily responsible for cytoskeleton remodeling. Activation of ROCK leads to the phosphorylation of serine in vimentin, resulting in partial disorganization and increased cellular motility [102,103,104].

The PKC/PKA pathway members interact with integrins, cell adhesion molecules, and IFs, ensuring their overall involvement in cellular processes. The kinases in this pathway phosphorylate vimentin filaments and regulate their interactions with actin and microtubule structures, allowing for dynamic adaptation to internal cellular changes [105,106].

#### 2.3.4. Functions of Intermediate Filaments

The key function of this component of the cytoskeleton is to provide mechanical elasticity to the cell as a scaffold. Thus, the cell is a dynamic, elastic structure that can resist stretching, shearing, and compression. Any disruption in the structure of IFs leads to increased cell fragility [8]. In addition, IFs regulate the position of organelles and maintain the intracellular architecture, which is particularly important for the axonal–dendritic architecture of neurons [96,107].

In the last decade, it has also been demonstrated that, due to their constant assembly and/or remodeling within the cell, IFs actively participate in processes such as cell adhesion and migration [108,109]. Furthermore, one of the most important functions of the cell, which ensures its survival, is its response to cellular stress, and stabilization of the cytoskeleton and modulation of signaling cascades (MAPK, PKC) are crucial components of this response [110].

Mechanical and oxidative stress leads to structural destabilization of the cytoplasm, which is controlled by the function of IFs [111,112]. For example, activation of the MAPK/p38 cascade during cellular stress leads to phosphorylation of GFAP and neurofilaments. As a result, the intermediate network is reorganized, and the cell adapts better to stressful conditions [113].

In addition to maintaining the mechanical elasticity of the cell, IFs play a role in coordinating intracellular transport, vesicle distribution, and cell adhesion. These functions are multifaceted, as desmin molecules determine the morphology and distribution of mitochondria, peripherin determines their retrograde transport, and vimentin enhances mobility and reduces membrane potential [114]. Additionally, the organization and dynamics of the Golgi apparatus are regulated by vimentin [115]. IFs can directly bind organelles through adapter proteins, but they can also influence each other through mutual signaling.

Cellular differentiation processes are induced and regulated through various intracellular signaling pathways. For example, IFs act as a structural framework for signaling molecules in the Notch, Wnt, YAP, TAZ, and TGF-β pathways [115,116,117,118,119]. Cellular stress signaling pathways also utilize IFs, as they can interact with cell stress-activated kinases and regulate their activity. Additionally, the ability to transmit intracellular signals and regulate the mechanical elasticity of cells allows for the active and efficient use of IFs in migration and proliferation processes.

Mechanical stress induces interactions between vimentin and keratins with focal adhesion molecules, thereby regulating cell adhesion. During cell migration, there are relatively rapid changes in cell morphology, and these rapid and significant changes are relayed by the intermediate filament network [115]. During cell migration and invasion into surrounding tissues, significant cellular deformations can occur. The structural integrity is maintained by the cytoskeleton as a whole, but IFs predominate in the mechanotransduction of the cytoplasm and prevent cell death during severe deformation, protecting the cell from rupture [120].

Recently, the role of IFs in the formation of vesicular transport has been described in detail. The absorption of liquids and macromolecules by cells is achieved through a process called endocytosis, which involves the formation of intracellular vesicles. It has been shown that IFs (vimentin and desmin) interact with early endosome sorting and recycling proteins (Rab), but the functional significance of these interactions remains unknown [121].

## 3. Main Stages of Corticogenesis and Involvement of Cytoskeleton Components

### 3.1. Proliferation of Neuronal Progenitors

Proliferation is one of the most crucial steps in cerebral cortex development, which is when neuronal precursors proliferate and subsequently differentiate into neurons and neuroglial cells. The primary role at this stage is to determine the final size of the brain, as the number of neuronal precursors determines the final number of mature neurons [122]. Thus, disruptions at this stage of neurogenesis lead to pathologies characterized by changes in the number of neurons and, consequently, brain size, manifested in micro- and macrocephaly [123].

Cytoskeletal components, primarily MTs and AFs, play a key role in ensuring the correct division of neuronal progenitors, since they determine the mechanical properties of the cell, the orientation of the mitotic spindle, the accuracy of chromosome segregation, and the completion of cytokinesis, which is ultimately critical for maintaining the pool of progenitors and normal development of the nervous system.

#### 3.1.1. The Role of Microtubules in the Proliferation of Neuronal Progenitors

MTs play a crucial role in the division of neuronal precursors; in particular, it ensures the assembly of the mitotic spindle and the establishment of the spindle axis; it also determines whether the division will be symmetrical (proliferative) or asymmetrical (differentiating).

As in other eukaryotic cells, the MTs that form the mitotic spindle in neuronal precursors are divided into three main groups: (1) kinetochore, whose main function is to pull sister chromatids to the poles of the cell; (2) astral, involved in the capture of chromosomes and the orientation of the mitotic spindle; and (3) interpolar, located between the poles and maintains the shape of the mitotic spindle [124].

Kinetochore MTs ensure adequate segregation of sister chromatids between daughter cells by depolymerizing them at the plus end in the kinetochore region [125]. This shortens the MTs and pulls the chromosomes toward the cell poles.

In addition to chromatid segregation, MTs control the spindle axis, which is determined by how the MTs are organized, anchored, and subjected to forces from the cell cortex. Astral MTs, anchored by their plus ends in the cell cortex, bind to the spindle with their minus ends [126]. Thus, the division axis in neuronal precursors can have a parallel orientation, which leads to symmetrical division and, consequently, to the formation of two stem cells, or a perpendicular orientation, leading, in turn, to asymmetrical division and the formation of a stem cell and a differentiating neuron [127]. Dyneins play an important role in axis orientation, creating pulling forces directed from the end of the microtubule toward the cortex, thereby rotating the spindle [128].

It should also be noted that at different phases of the cell cycle, MTs provide structural support to neuronal precursors, forming a dynamic internal framework that allows the cell to radically change shape without losing mechanical integrity, polarity, and division orientation [129].

#### 3.1.2. The Role of Actin Filaments in the Proliferation of Neuronal Progenitors

In addition to MTs, the proliferation of neuronal progenitors is also regulated by the actin component of the cytoskeleton. AFs control cell rounding during mitosis, participate in the regulation of cell cycle progression, and, together with MTs, maintain the orientation of the mitotic spindle, thereby ensuring the asymmetry of neuronal progenitor division.

Filamin A, an actin-binding protein, has been shown to control the proliferation of neuronal progenitors by regulating the duration of the G2/M phase. Loss of this actin-binding protein results in cell cycle prolongation and a decrease in proliferation, likely due to disruption of actin filament rearrangement, as demonstrated in *in vivo* experiments in mice [130].

When a cell enters the G2/M phase, the actin cytoskeleton undergoes remodeling, which is necessary to change the cell’s shape from elongated to rounded [131]. This process forms a dense network of fibrillar actin (F-actin), located directly beneath the plasma membrane. This creates a tense cortical membrane. The rounded cell shape plays a critical role in maintaining the symmetry of the mitotic spindle [132].

Subsequent processes play a critical role in ensuring the asymmetric division of neuronal progenitor cells. AFs regulate cortical tension through asymmetric polymerization. This creates an uneven tension between the apical and basal sides of the cell, leading to rotation of the mitotic spindle toward the side of lesser resistance and alignment of the division axis [133,134]. Thus, impaired F-actin remodeling leads to rounding defects, which in turn disrupt the formation and orientation of the mitotic spindle and, consequently, lead to improper chromosome segregation [135].

During the final phase of cell division—cytokinesis—a constriction, known as the cleavage furrow, forms at the cell equator due to the retraction of the cell equator by AFs [136]. F-actin polymerizes and, together with myosin, forms a contractile ring, which ensures the proper separation of the daughter cells. Thus, F-actin polymerization plays a crucial role during the assembly, disassembly, and contraction of the contractile ring [137].

#### 3.1.3. The Role of Intermediate Filaments in the Proliferation of Neuronal Progenitors

Although IFs are not directly involved in chromosome segregation, spindle orientation, or other active processes, they also play a role in the proliferation of neuronal progenitors. Their primary function is to mechanically stabilize cell shape and, therefore, reduce the likelihood of mechanically induced cell division errors [8,115].

When cells enter mitosis, IFs depolymerize, forming cytoplasmic aggregates of disassembled IFs, and their constituent proteins, vimentin and desmin, are hyperphosphorylated at several specific sites [138]. This temporarily loosens the intermediate filament network, and the cell becomes more flexible, but its mechanical strength is not lost.

In the cytokinesis phase, the network of IFs is gradually restored, and they are predominantly localized in the region of the cleavage furrow, participating in maintaining the cell shape [115] and regulating the contraction of the contractile ring [139].

**Figure 1:**
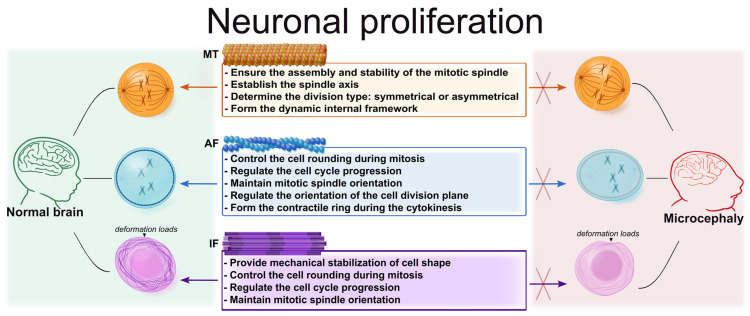
The role of cytoskeletal elements in the proliferation of neural progenitors: MTs—microtubules; AFs—actin filaments; IFs—intermediate filaments. MTs form the mitotic spindle and determine its axis, ensuring the separation of chromatids and creating a dynamic cell framework. AFs also maintain the orientation of the spindle axis and, together with MTs, determine the type of cell division (symmetrical/asymmetrical). Additionally, AFs control the duration of cell cycle phases and determine the cell shape, which is particularly important in different types of division. The final stage of cytokinesis is also dependent on AFs, which form the contractile ring. IFs, on the other hand, provide mechanical support to the cell. Disruptions in these functions of cytoskeletal elements lead to defects in the proliferation of neural precursor cells and the subsequent development of microcephaly.

Thus, the process of neuronal progenitor proliferation depends on the coordinated interaction of all cytoskeletal elements, each of which contributes to various stages of cell division (Figure 1). MTs ensure the assembly and stability of the mitotic spindle, thereby providing accurate chromosome segregation, while AFs determine the mechanical properties of the cell, participating in cell rounding upon entry into mitosis, the orientation of the division plane, and the formation of the contractile apparatus during cytokinesis. IFs, in turn, maintain the mechanical integrity of neuronal progenitors, reducing the sensitivity of dividing cells to deformation loads in the developing nervous tissue.

### 3.2. Neuronal Migration

Neuronal migration is a critical stage in the embryonic development of the CNS, involving the movement of newborn neurons to their destination. During the development of the cerebral cortex, newborn neurons undergo radial migration, moving along the processes of radial glia from the ventricular zone (VZ) to the cortical plate (CP) [140]. As cortical neurons proceed in their development, they change their shape, first transitioning from a bipolar to a multipolar state, and then back to a bipolar state to commence migration [141]. This process is critical for the proper formation of the layered architecture of the cortex, and its disturbances are associated with pathologies such as lissencephaly, polymicrogyria, and some forms of autism [142,143]. In addition, interneurons migrate via the tangential mode, characterized by a move parallel to the brain surface independently of the radial glial cells [144].

Migration is a multi-stage process that relies on dynamic organization of the cytoskeleton; it is highly dependent on the coordinated work of MTs and the actin cytoskeleton [140].

#### 3.2.1. The Role of Microtubules in Neuronal Migration

Before migration initiation, a neuron forms a leading process, which determines the direction of movement. Its stabilization and rigidity are maintained by MTs, which extend from the centrosome toward the process tip and form its supporting framework [145]. Thus, MTs provide mechanical support for the leading process, preventing its fragility and curvature. Furthermore, microtubule growth facilitates the elongation of the leading process before the neuron’s soma begins to migrate. Specifically, MTs are captured at their plus end by a network of AFs in the cell cortex and are pulled in as the process elongates [146].

One of the most important functions of MTs at this phase of neurogenesis is the traction of the cell nucleus (nucleokinesis) toward the leading process, which is achieved due to interaction with motor proteins [145]. The microtubule motor proteins dynein and kinesin interact with the nucleus through the LINC complex and move the nucleus along polarized MTs [147].

A recent study demonstrated that the centrosome, the most important MTOC in the cell, is critical for radial neuronal migration [148]. It was found that altered γ-tubulin activity in the centrosome disrupts the cyclical formation of the cytoplasmic expansion in front of the nucleus, which is necessary for successful neuronal migration to the CP.

MTs play an important role in transport, as motor proteins carry various vesicles, receptors, and adhesion proteins along with them. These proteins are essential at the leading edge of the cell for navigation and response to external stimuli during migration. In particular, the importance of cargo transport along MTs for migration was demonstrated in a study on zebrafish [149]. The study demonstrated that disruption of cargo transport along MTs disrupts the migration process to the same extent as disruption of endosome formation or changes in the Golgi apparatus.

MTs do not act in isolation; they interact with the actin component of the cytoskeleton, the action of which will be discussed below. Specifically, AFs located at the leading edge of the cell pull the MTs forward, and mechanical interactions between actin and MTs ensure coordinated cell movement [146].

#### 3.2.2. The Role of Actin Filaments in the Process of Neuronal Migration

AFs, in turn, control the dynamics of the leading edge of the cell, participate in the formation of the leading process, namely lamellipodia and filopodia, regulate cell repulsion and participate in the contraction of the posterior part of the cell [140,150].

The F-actin network is located predominantly at the leading edge of the migrating neuron, in the region of the leading process, where its polymerization predominates [151]. Active actin polymerization promotes the formation of lamellipodia, which are wide-mesh structures that push the cell membrane forward, as well as filopodia, which are thin projections that perceive external signals. When actin polymerization is disrupted, neuronal migration becomes impossible [135]. Depolymerization of AFs, in turn, predominates at the trailing edge, which ensures the compression and retraction of this part of the cell.

Actin remodeling also plays a crucial role in nucleokinesis, although this process primarily depends on the microtubule component. During radial migration, MTs, together with dynein, initiate nuclear movement, facilitating its pull toward the cell’s leading edge. Actin, in turn, works with myosin to propel the nucleus forward [145]. Thus, proper nuclear movement requires the combined action of these two cytoskeletal components.

#### 3.2.3. The Role of Intermediate Filaments in Neuronal Migration

IFs are less involved in the migration process as compared to AFs and MTs. Although they lack motor dynamics, they perform critical mechanical and organizational functions. During migration, the leading process of the neuron is formed by actin polymerization; the nucleus and cell soma move along the microtubule network, while IFs provide mechanical support and regulate movement [2,150,152]. During nucleokinesis, IFs maintain the shape of the soma as the nucleus moves, thereby allowing the cell to be simultaneously rigid and elastic [153].

**Figure 2:**
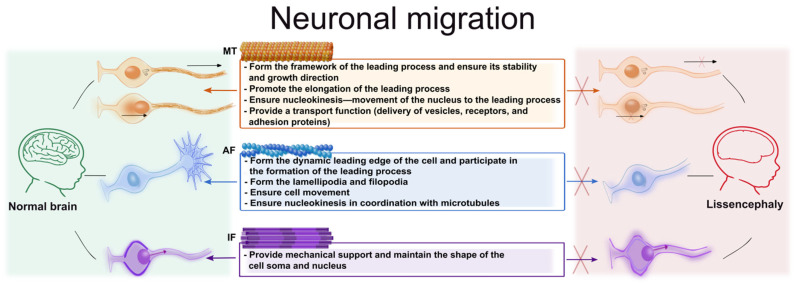
The role of cytoskeletal elements in the neuronal migration: MTs—microtubules; AFs—actin filaments; IFs—intermediate filaments. MTs extend from the centrosome to the growth cone, determining the stability and rigidity of the leading edge, while MTs polymerization promotes its growth. MTs also ensure nucleokinesis towards the growing leading edge. Polymerization of AFs in the front of the cell determines the growth of the leading edge, filopodia, and lamellipodia, while its depolymerization in the back of the cell provides its retraction. At the same time, IFs maintain the shape of the cell, determining its rigidity and elasticity through mechanical support. Defects in cytoskeletal components during neuronal migration lead to the development of lissencephaly.

Thus, the combined and well-coordinated work of the three components of the cytoskeleton allows newborn neurons to effectively move in a directed manner in space, reaching their final position, which is critically important for the correct formation of the layered structure of the cortex (Figure 2).

### 3.3. Neuronal Polarization

Neuronal polarity is the asymmetric organization of cells, forming a single axon and multiple dendrites. These processes have different functions, structures, and molecular contents. Neuronal polarity establishment is a key stage of neurodevelopment, determining the direction of signal transmission and the integration of neurons into networks. Of the several neurites, only one is selected to become the axon. This choice and subsequent polarity are dependent on the cytoskeleton, its reorganization, and the interaction of cytoskeletal components [154].

Neuronal polarization is accompanied by a redistribution of the actin cytoskeleton in the growth cone and the axial orientation of MTs [155]. IFs (nestin, vimentin) ensure the formation of processes and stabilization [152].

#### 3.3.1. The Role of Microtubules in Neuronal Polarization

Axonal identity is achieved through the coordinated functioning of cytoskeletal components, with MTs playing a key role in this process. MTs themselves are polar structures, containing plus and minus ends, which allow them to organize the correct direction of neurite transport and growth [156]. Thus, the axon is characterized by a unipolar orientation of MTs, with the plus ends directed distally. The remaining neurites—dendrites—are characterized by a mixed organization of MTs [157]. Selective stabilization of MTs in one specific neurite leads to its subsequent specification. This is achieved through the accumulation of stabilized MTs and enhanced microtubule-dependent transport [155].

Thus, even before the visible axon appears, localized microtubule stabilization is detected in one of the neurites. This process determines whether a given neurite will become an axon, as demonstrated in *in vitro* experiments on neuronal cultures [158]. Increased microtubule stability leads to the formation of multiple axons.

Furthermore, process specification is regulated by microtubule motility within neurons. MTs have been shown to move retrogradely within neurites toward the soma, preventing the accumulation of kinesin-1 protein in neurites. However, in one neurite, this flow slows sharply, leading to increased kinesis-dependent axonal transport and axonal specification of that particular neurite [159].

Once an axon’s fate has been determined, the axon must maintain its specification throughout its life. This is achieved through stable microtubule organization, the maintenance of kinesin/dynein transport, and the formation of an actin filter at the axon’s initial segment, which prevents dendritic proteins from entering the axon [160,161].

#### 3.3.2. The Role of Actin Filaments in Neuronal Polarization

AFs, unlike MTs, primarily play an inhibitory role in axonal specification. The actin cytoskeleton is a dynamic structure, and when the actin filament network in one neurite becomes less organized and stable, this reduces the obstacle to MTs and promotes their polarization and growth in that process [162]. Thus, the neurite with the lowest actin density is more likely to become an axon, and local depolymerization of F-actin promotes microtubule penetration into the neurite [163].

Furthermore, the study shows the presence of spontaneously occurring actin polymerization cascades—actin waves—progressing from the cell body to the tips of neurites. The emergence of actin waves has been shown to be characteristic of the neurites of multipolar neurons, as well as of future axons [164].

#### 3.3.3. The Role of Intermediate Filaments in Neuronal Polarization

The role of IFs, as opposed to MTs and AFs, remained largely unexplored; however, current data demonstrate that IFs contribute to the maintenance of stable neuronal polarity. Specifically, neurofilaments stabilize the axon, maintain its size, and participate in the long-term maintenance of polarity and specification [96].

It has also been shown that IFs proteins, in particular, nestin, are involved in the regulation of signaling pathways that affect other components of the cytoskeleton, indirectly regulating the polarization of developing neurons [165].

**Figure 3:**
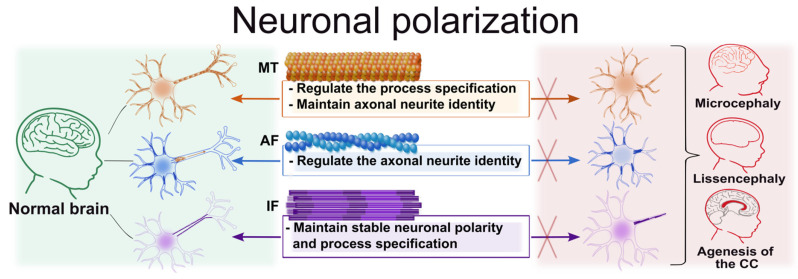
The role of cytoskeletal elements in the neuronal polarization: MTs—microtubules; AFs—actin filaments; IFs—intermediate filaments; CC—corpus callosum. The stabilization of MTs in a particular neurite determines its specification. The microtubule organization in the axon is characterized by prominent polarity, with the plus ends directed distally, in contrast to dendrites, where there is no strict organization. The destabilization of the AFs in the neurite contributes to its axonal specification, while the formation of an actin filter prevents the entry of dendritic proteins into the axon. IFs, in turn, stabilize the axon, determining its size and participating in the long-term maintenance of polarity and specification. Disruptions in neuronal polarization due to improper cytoskeletal function can lead to various pathologies, including microcephaly, lissencephaly, and agenesis of the corpus callosum.

Thus, neuronal polarization is the result of the coordinated work of all cytoskeletal components (Figure 3). AFs determine the formation of primary neurites, MTs ensure the transport and stabilization of axonal process specification, and IFs promote long-term structural stability of established neuronal polarity.

### 3.4. Growth of Axons and Dendrites

Neurite outgrowth is the process of nerve cell process elongation, critical for the formation of neural networks. The growth cone, a dynamic structure located at the end of a process (axon or dendrite) responsible for advancement and navigation, plays a key role in this process. The growth cone perceives chemotactic signals of repulsion or attraction and, depending on the stimulus received, converts the external signal into changes in the cytoskeleton, which determines the growth vector of the process. The growth cone responds to external chemical signals (netrins, semaphorins, ephrins) through their respective receptors. The activation of the receptors transmits the signal to calcium-dependent signaling, Rho GTPase cascades, kinases, and other signaling pathways [166].

The growth cone is divided into three spatial domains: the peripheral (P-domain), which is rich in actin and contains filopodia and lamellipodia on the membrane surface, the transitional (T-domain), which is defined as the interaction zone of actin networks and MTs, and the last zone, the central (C-domain), in which stable MTs dominate, and organelles and vesicles are also present [167].

The classical growth cone model describes its growth in three stages: polymerization elongation of filopodia and lamellipodia due to actin, advancement of MTs and translocation of organelles into the expanded growth cone, stabilization of the formed axon segment, followed by continued growth [166].

#### 3.4.1. The Role of Microtubules in Neurite Outgrowth

MTs provide the structural and transport framework for growing neurites. In growing processes, they form a directed network of bundles oriented along the growth cone axis and serve as “rails” for retrograde and anterograde motor transport [168]. In axonal processes, MTs are predominantly oriented with their plus ends toward the distal end, thereby ensuring directed kinesin-dependent transport of molecules necessary for axon growth [169]. Unlike axons, dendrites are characterized by a mixed orientation of MTs, which ensures their more branched growth pattern [170].

The dynamic instability of MTs ensures their penetration into the growth cone and supports neurite elongation. Thus, kinesin-dependent movement of MTs relative to one another ensures the initial elongation of the process [171]. Some MTs penetrate filopodia, promoting directed growth of the cone by interacting with the actin network. Proteins at the ends of MTs link the plus ends to actin structures, ensuring the joint coordination of the two cytoskeletal networks [167].

Furthermore, it has been shown that neurites differ in the degree of microtubule organization. Axons contain more stable MTs with a higher proportion of modified tubulins, which enables the growth of a process over longer distances. Dendrites, on the other hand, are characterized by less stable MTs that undergo reorganization more frequently, which is also necessary for their branching [172].

#### 3.4.2. The Role of Actin Filaments in Neurite Outgrowth

The actin cytoskeleton is the primary generator of the driving force during axon and dendrite growth, particularly at their distal ends. In the peripheral domain, F-actin forms filopodia and lamellipodia, responsible for “sensing” the area around the growth complex [173].

Actin also creates a dynamic actin flow, which underlies the mechanism of neurite elongation. In the region of the plus ends located closer to the membrane, G-actin polymerizes into F-actin, while in the more proximal region, it depolymerizes. Thus, actin polymerization leads to membrane forward movement and neurite growth, and its remodeling allows the growth cone to change direction in response to navigation signals [173]. Actin-dependent retrograde transport is also important, regulating the balance between growth cone advancement and retreat [166].

It has also been demonstrated that F-actin ensures not only the elongation of neurites, but also their branching due to the formation of “actin patches” [174].

#### 3.4.3. The Role of Intermediate Filaments in Neurite Outgrowth

IFs, unlike other cytoskeletal components, do not directly generate movement, but provide mechanical stability to growing processes, performing a supporting and stabilizing role. In particular, their role increases as neurites grow.

In developing neurites, nestin and vimentin ensure cytoskeletal plasticity; disruption of their functions leads to suppression of neurite outgrowth, as demonstrated *in vitro* [175]. During subsequent axon and dendrite growth, these proteins are replaced by neurofilaments, which accumulate in neurites, providing their mechanical stability and maintaining their elasticity as their length increases [152].

Differences in the structure and organization of IFs in axons and dendrites are noteworthy. In axons, neurofilaments form stable, dense longitudinal structures that provide stability to the process [176]. Dendrites, in turn, are characterized by a less dense IFs network capable of rapid reorganization, which determines its importance for active branching [152].

**Figure 4:**
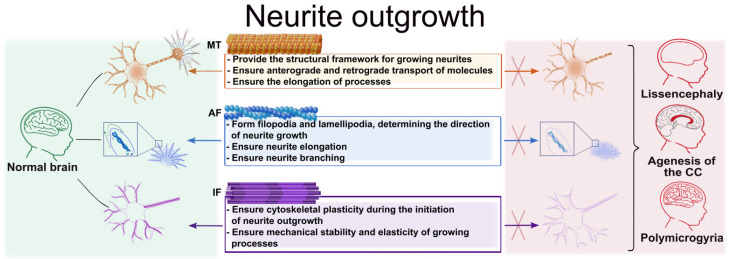
The role of cytoskeletal elements in neurite outgrowth: MTs—microtubules; AFs—actin filaments; IFs—intermediate filaments; CC—corpus callosum. The large length of the axon is determined by the stabilization of MTs. Their dynamic properties and mixed organization allow the dendrites to be more branched. MTs penetrate the growth cone and support the elongation of the neurite, and some MTs penetrate the filopodia and interact with the AFs to ensure the directional movement of the growth cone. In the peripheral region of the growth cone, the AFs primarily form filopodia and lamellipodia. As the AFs approach the membrane, they polymerize while simultaneously depolymerizing in the proximal region, which contributes to the elongation of the neurites and sets the direction of the growth cone. In the axon, the IFs provide stability by forming stable, dense structures, while in the dendrites, the IFs form a less dense network, allowing for active branching. Defects in the cytoskeletal processes that mediate neurite growth can lead to malformations, such as lissencephaly, agenesis of the corpus callosum, and polymicrogyria.

Thus, axon and dendritic growth is ensured by the coordinated interaction of all cytoskeletal components (Figure 4). AFs shape the dynamic architecture of the growth cone and determine the direction of neurite progression through the formation of filopodia and lamellipodia. MTs support the elongation of the process, serving as its structural framework, and also facilitate the transport of proteins and organelles. IFs, in turn, provide mechanical stability to the developing processes and contribute to their stabilization.

### 3.5. Synapse Formation and Plasticity

Synapses are specialized junctions between cells that facilitate signal transmission. The formation and plasticity of synapses underlie the development of neural networks, which must possess a high degree of structural organization and the ability to rapidly rewire neuronal connections.

The cytoskeleton plays a central role in these processes, providing a structural framework for axonal growth cones and dendritic spines, intracellular transport, and adaptation of synaptic architecture in response to neuronal activity. Resent research shows that cytoskeletal elements not only perform a supporting function but also actively participate in the regulation of synaptogenesis and activity-dependent plasticity, coordinating local structural changes with molecular signaling cascades at the pre- and postsynaptic levels.

#### 3.5.1. The Role of Microtubules During Synapse Formation and Plasticity

MTs were initially considered as “transport pathways” used to deliver proteins and organelles to neurites. However, recent data show that the microtubule component of the cytoskeleton actively participates in the formation, organization, and dynamic regulation of synaptic contacts on both the presynaptic and postsynaptic sides [177].

MTs ensure the delivery of presynaptic components (receptors, regulatory proteins, vesicles) to synaptic contacts, especially during periods of increased activity [178]. Presynaptic MTs participate in the organization of synaptic terminals by controlling the distribution of synaptic vesicles (SVs), which depends on microtubule-mediated transport. This is particularly true through motor proteins of the kinesin family, which transport SVs to the active zone and maintain their population within the synapse [179].

Furthermore, MTs also play a role in spine-mediated synaptic plasticity. MTs can grow from the dendritic shaft into the spine itself, particularly in response to synaptic activity, such as NMDAR activation and Ca^2+^ influx [180]. Microtubule activity within dendritic spines is actin-dependent and regulated by proteins that ensure the capture of the plus ends of MTs [181]. Microtubule activity in this context creates a pathway for the kinesin-mediated transport of vesicles and regulatory proteins into the postsynapse [180].

#### 3.5.2. The Role of Actin Filaments During Synapse Formation and Plasticity

One of the main structures important for signal transmission are dendritic spines, responsible for synaptic plasticity such as long-term potentiation (LTP) and long-term depression (LTD). The main structural and dynamic components of dendritic spines are AFs. The formation and stability of spines depend on actin dynamics: spine size and dynamics are controlled through polymerization and depolymerization of actin structures [182]. These processes are controlled by actin-binding proteins (cofilin, profilin, Arp2/3) [183].

F-actin remodeling is triggered by synaptic activity. In particular, during the induction of LTP, NMDA receptor-mediated Ca^2+^ influx occurs, triggering signaling cascades (e.g., Rho GTPase), leading to actin polymerization and an increase in spine size, and, consequently, to a strengthening of the synaptic connection [184]. Binding of the motor protein myosin II to F-actin also occurs, which is necessary for the stabilization of LTP [185]. LTD, in turn, is accompanied by a tendency toward actin depolymerization and a decrease in spine size.

#### 3.5.3. The Role of Intermediate Filaments During Synapse Formation and Plasticity

Previously, IFs were considered solely structural elements, providing mechanical stability and axon diameter by forming dense bundles and stabilizing organelles. However, it was demonstrated that specific assemblies of neurofilament proteins are found directly at synapses, particularly in the postsynaptic regions of dendritic spines [186].

Neurofilaments are localized near postsynaptic densities and associate with receptors, suggesting their potential influence on receptor organization, which is necessary for the formation of stable synapses [187]. Regulation of receptor localization is necessary for synaptic plasticity in response to activity, particularly LTP and LTD [188]. Deficiencies in neurofilament proteins, particularly NF-L, are associated with a decrease in the number and density of dendritic spines, demonstrating the necessity of neurofilaments for synaptic plasticity [188].

In addition, synapses often contain short structures (protofilaments) rather than the long bundles characteristic of axonal processes, reflecting the specific organization of IFs in the synaptic compartment [186].

**Figure 5:**
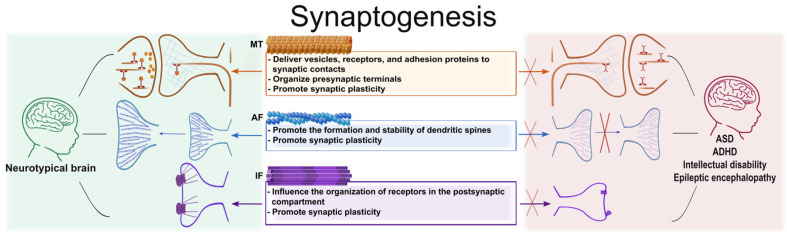
The role of cytoskeletal components in synapse formation: MTs—microtubules; AFs—actin filaments; IFs—intermediate filaments; ASD—autism spectrum disorder; ADHD—attention deficit hyperactivity disorder. MTs shape and modulate terminal dynamics and synaptic plasticity. MTs also cooperate with kinesin molecules to deliver vesicles and components to pre- and postsynaptic terminals. AFs determine and control spine size and dynamics. IFs concentrate near the postsynaptic terminal and organize receptors. Disturbances in the cytoskeletal elements that mediate synaptogenesis lead to ASD, ADHD, and epileptic encephalopathies.

Thus, it can be concluded that synaptogenesis and subsequent synaptic plasticity are impossible without the coordinated functioning of all cytoskeletal elements (Figure 5). MTs organize intracellular transport and contribute to the stabilization of synaptic contacts. The actin component of the cytoskeleton, in turn, determines the shape and size of dendritic spines, regulating their remodeling in response to changes in neuronal activity. IFs perform both a supporting function and regulate the organization of receptors in the postsynaptic region, which is necessary for effective signal transmission.

## 4. NDDs Associated with Dysregulation of Cytoskeleton

Cytoskeletal dysregulation is considered to be a key molecular mechanism underlying a range of NDDs. In this chapter, we focus on microcephaly, lissencephaly, dysgenesis of the corpus callosum, and synaptopathies—conditions often associated with defects in cytoskeletal components or the proteins that control their dynamics. The development of these pathologies is associated with disruptions at various stages of neurogenesis—from the proliferation of neuronal progenitors to migration, neurite outgrowth, and synaptic contact formation. We summarize information on how pathogenic variants in genes encoding cytoskeletal structural elements or their regulators lead to specific cellular defects in neurogenesis and the development of characteristic clinical features. We included in the review genes for which a link with cytoskeletal components has been demonstrated, the molecular mechanism of action has been described, and convincing evidence of a relationship with pathology has been provided—at the level of clinical observations and experimental models.

### 4.1. Microcephaly

Microcephaly is a clinical condition characterized by a reduction in the occipital–frontal head circumference of more than two standard deviations (SDs) from the population mean for a given age and gender [189]. It occurs with a frequency of 1.5 to 8.7 per 10,000 births in Europe and the United States, respectively [190,191]. In addition to reduced head size, patients with microcephaly may also experience intellectual disability, developmental delay, epilepsy, and cerebral palsy [192].

Depending on the time of manifestation, microcephaly is divided into primary (congenital) and secondary (postnatal). While primary microcephaly is due to decreased production of neural progenitors or increased cell death, secondary microcephaly is usually associated with impaired neurito- or synaptogenesis [192,193]. The widespread use of next-generation sequencing has led to the identification of numerous genes whose mutations cause this pathology. Currently, the Human Phenotype Ontology (HPO) (v2.1.2) database contains 1413 genes associated with microcephaly (HP:0000252) [194].

These include genes that control cell cycle, ribosome and centrosome biogenesis, DNA repair, mitotic spindle formation, chromatin condensation, Wnt/β-catenin signaling, and nuclear envelope structure [195,196]. Mutations in genes encoding various cytoskeletal proteins also play a significant role in the pathogenesis of microcephaly.

There is now increasing evidence that defects in the dynamics and regulation of MTs may underlie a wide range of neurodevelopmental disorders, including microcephaly. The pathogenic variants in the genes encoding tubulins, the main structural components of the cytoskeleton, may cause the reduced brain size.

For example, pathogenic variants of the *TUBB5* gene (M299V, V353I, and E401K), that encodes β-tubulin 5, have been identified in four unrelated patients with microcephaly [197,198]. Indeed, *Tubb5* is one of the most highly expressed tubulin isoforms in the embryonic mouse brain. It is detected in aRGCs, intermediate progenitors, migrating neurons, and postmitotic neurons. A similar phenotype was also observed in a mouse model with conditional knockout of *Tubb5* as well as in E401K substitution homozygous animals [199]. Knockdown of this gene in the embryonic brain of mice at the E14.5 stage resulted in a significant decrease in the number of cells in the CP and an increase in the number of cells in the VZ and intermediate zone (IZ). In addition, in utero electroporation of genetic constructs that overexpress mutant forms of *TUBB5* (p.Met299Val, p.Val353Ile, and p.Glu401Lys) into the developing mouse brain led to an accumulation of cells in the IZ and a depletion of cells in the CP [197]. Furthermore, *Tubb1*-deficient mice display a significant induction of apoptosis [199].

PRUNE1, a member of the DHH (Asp-His-His) superfamily, plays an important role in the regulation of MTs and has exopolyphosphatase and phosphodiesterase activities [200,201]. PRUNE1 can also interact with several intracellular proteins involved in the regulation of the cytoskeleton, including Nm23-H1/H2, GSK-3β, and α- and β-tubulins [200]. These protein–protein interactions may play an important role in cell division and migration during brain development, which is consistent with the high levels of *Prune1* expression in the cortex, hippocampus, midbrain, and cerebellum during early embryonic development [202].

A link was found between different variants of the *PRUNE1* gene and the development of microcephaly [203,204,205]. The p.Asp30Asn and p.Arg297Trp variants were associated with reduced cell proliferation, impaired migration, and differentiation. One possible cause of these cellular defects is the dysregulation of MTs, as these mutations have been shown to disrupt the polymerization of this cytoskeletal component. SH-SY5Y cells expressing the mutant protein were found to have shorter MTs compared to wild-type cells [204].

A recent study identified another gene that is critical for the normal functioning of the microtubules during neurodevelopment, namely *SPOUT1*, also known as *CENP32* or *C9ORF114*. This protein is a SPOUT-domain-containing RNA methyltransferase that plays a role in post-transcriptional RNA modification, as well as in the organization of the mitotic spindle and the proper segregation of chromosomes during cell division [206,207]. *SPOUT1* is highly expressed both during embryonic development and postnatally, and is widely distributed in various human tissues and organs [208].

Patients with mutations in the *SPOUT1* gene have characteristic clinical features designated as the autosomal recessive neurodevelopmental disorder SpADMiSS (SPOUT1 Associated Development delay Microcephaly Seizures Short stature) [207,208]. In experiments involving the knockout of this gene in zebrafish and mice, the phenotype observed in patients was reproduced.

For example, these animals showed impaired progression of the cell cycle, accompanied by increased apoptosis of neural progenitors, which was also confirmed in mice with temporary knockdown of *Spout1* in cortical stem cells. Additionally, depletion of this gene in U2OS cells using siRNA led to the displacement of centrosomes from the spindle poles towards the metaphase plate, as well as increased micronucleus formation. Although these abnormalities are associated with reduced RNA–methyltransferase activity of *SPOUT1*, the exact mechanisms underlying mitotic spindle disorganization and dysfunction remain to be elucidated [207].

Heterozygous mutations in the *NIN* gene cause Seckel syndrome type 7 (OMIM 614851), which is characterized by severe microcephaly, facial dysmorphism, and other abnormalities [209]. Dauber et al. described two sisters with the phenotype of this syndrome, and Sanger sequencing revealed heterozygous mutations in the *NIN* gene for two missense variants: p.Gln1222Arg and p.Asn1709Ser [210]. Knockdown of this gene in zebrafish resulted in the phenotype of microcephalic primordial dwarfism [210]. Ninein is a centrosomal protein that binds to the mother centriole during asymmetric cell division and can act as a maturation factor for centrosomes [211,212,213,214]. It has also been shown that ninein is a coactivator and adapter for the dynein–dynactin complex [215]. Ninein plays a crucial role in the nucleation of microtubules and the attachment of microtubules to the mother centriole [216,217]. Disruption of ninein expression reduces the nucleation of microtubules, leading to a defective organization of the radial network of microtubules, and the disruption of ninein localization and activity is associated with defects in non-centrosomal arrays of microtubules in neurons [218,219,220].

Along with MTs, the actin cytoskeleton also plays an important role in brain development. In recent years, there has been an increasing body of evidence linking mutations in various genes encoding the actin cytoskeleton proteins to the development of microcephaly. *MTSS2*, also known as *MTSS1L*, is highly expressed in aRGCs in humans and plays a role in generating negative membrane curvature, which is essential for the formation of plasmalemmal projections [221].

Microcephaly was diagnosed in several patients with a heterozygous missense mutation 2011C>T (p.Arg671Trp; R671W) in the *MTSS2* gene [221,222]. Knockdown of this gene in mouse neural progenitor cells demonstrated accumulation of cells in the VZ, improperly oriented apical processes of aRGC, a decrease in the number of aRGC, and an impairment in the cell cycle progression. Additionally, when the expression of *Mtss2* was suppressed in C6 rat glioma cells, a pronounced mitotic block was observed [221].

Microcephaly can also be part of a more complex set of disorders. For example, Baraitser–Winter syndrome (BRWS) is a rare genetic disease characterized by short stature, congenital ptosis, high eyebrow arches, hypertelorism, coloboma of the eyes, hearing loss and malformations of the brain, including lissencephaly [223], in addition to microcephaly. It is believed that the main causes of the development of BRWS are various mutations in the *ACTB* and *ACTG1* genes encoding β- and γ-actin, respectively [223,224,225,226,227].

Knockout mice for these genes do not accurately reproduce the cortical malformations associated with this syndrome in humans, but experiments with cerebral organoids derived from induced pluripotent stem cells (IPSCs) from patients with BRWS revealed reduced organoid size and a reduced aRGC pool compared to control samples [228].

### 4.2. Lissencephaly

Lissencephaly (from the Greek “lissos”—smooth, “kephale”—head/brain—“smooth brain”) is a genetically and phenotypically heterogeneous group of cortical malformations in which the normal formation of gyri and sulci is disrupted [229]. Lissencephaly is included in the category of rare diseases—the prevalence is about 1.2 cases per 100,000 live births [230].

This malformation is characterized by agyria/pachygyria or the formation of a subcortical “double cortex.” As a result, the surface of the cerebral cortex becomes flattened or has an abnormally wide sulci. Within the spectrum of lissencephaly, there are various forms, namely complete agyria, pachygyria, and subcortical striate heterotopia, in which some neurons remain outside the cortical structure [229,231].

This disorder develops as a result of impaired neuronal migration during embryogenesis between 12 and 24 weeks of pregnancy, which disrupts the normal organization of the cortex [230]. Patients with classical lissencephaly have motor impairments and intellectual disabilities, and hypotension often occurs at an early age, which then develops into spasticity of the limbs [232,233].

In some cases, there are additional structural abnormalities of the brain: agenesis of the corpus callosum, cerebellum, subcortical heterotopia, and heterogeneity of the cortical layers [234]. There are more than 20 types of lissencephaly, most of which are divided into two main categories: the most common classic lissencephaly (type 1) and cobblestone lissencephaly (type 2). Each category has similar clinical manifestations but differs in genetic cause [235,236].

Abnormal or delayed migration is a key factor that prevents the proper formation of the gyri and sulci of the cerebral cortex. This process is related to the need for dense packing of neurons and their proper polarization for the organization of cortical layers. In cases where migration is disrupted or delayed, the formation of characteristic folded structures such as gyri and sulci does not occur, leading to conditions such as hypogyria, agyria, and pachygyria. These pathologies are characterized by a smooth brain surface or the presence of numerous, wide, and sparse gyri [237,238]. If the migration process is less severe and neuronal functions are partially preserved, subcortical heterotopia may develop, which is the accumulation of neuronal cells outside their normal layered structures [239].

Mutations in the *LIS1* and *DCX* genes cause type 1 lissencephaly and account for approximately 85% of cases of classical lissencephaly [233]. This variant includes isolated lissencephaly and heterotopia of the subcortical cortex [239,240]. Both of these genes encode MAPs that are essential for neuronal differentiation and migration [241].

The *LIS1* gene (Lissencephaly 1; alternative name PAFAH1B1) encodes the LIS1 protein, which is an important regulator of the cytoskeleton and intracellular transport. Pathogenic variants of *LIS1* that disrupt the expression of this gene cause Miller–Dieker syndrome, which is characterized as a congenital brain malformation due to the deletion of a region on the short arm of chromosome 17 [239]. This region was shown to be genetically unstable, as a whole-exome sequencing study of 7678 patients with intellectual disabilities and other abnormalities revealed microdeletions and microduplications at locus 17p13.3 [242].

There are other confirmed cases of severe lissencephaly due to mutations in *LIS1* locus and functional loss of *LIS1* [243,244,245]. In addition to lissencephaly, mutations in this gene also cause hypoplasia of the corpus callosum and enlargement of the ventricles [239,246,247]. Early studies in mouse models with knockout of this gene revealed that homozygous mutations of *Lis1* were lethal for the animals [248]. The brains of heterozygous animals showed disorganization of the cortical layers [249,250].

The LIS1 protein is involved in the regulation of MTs dynamics as well as the function of motor proteins [251,252]. It can directly bind tubulin. On the other hand, it is involved in the control of the dynein/dynactin-mediated dynamics of MTs. The *LIS1* gene product can be defined as a microtubule stabilizer [253]. It seems that during neuronal migration, this protein is located in the centrosome region, and its primary function at this stage is to stabilize MTs and reduce the probability of MTs catastrophes [254].

In addition, due to its involvement in the stabilization of MTs, LIS1 is also involved in the regulation of cargo transport by dynein [255]. It has been shown that LIS1 deficiency leads to an increase in the perinuclear localization of MTs and a decrease in the distribution of microtubule plus ends at the cell periphery [237,256]. Another effect of the deficiency is the disruption of the interaction between the cell nucleus and centrosome, which results in a decrease in the distance between these structures during active neuronal migration [237,257].

It has been shown that there is a LIS1-CLIP1 complex that localizes to the plus ends of MT. It is worth noting that this combination is part of a larger structure that includes the small GTPases CDC42 and RAC1, which regulate actin. Thus, LIS1 promotes the polymerization of F-actin in the distal region of leading processes. Additionally, LIS1 acts as an activator of CDC42/RAC1 and an inhibitor of RhoA. A deficiency in LIS1 molecules leads to a decrease in the amount of F-actin in the anterior area of the migrating neuron, resulting in a size reduction in the filopodia required for neuronal migration [237,258].

In recent years, the mechanisms of LIS1 and dynactin binding have been described in more detail, as well as how LIS1 binds to dynactin and releases its autoinhibition, which promotes the assembly of the dynein–dynactin adapter complex [259]. It has been shown how specific mutations in *LIS1* or alterations in dynactin expression can disrupt nucleokinesis and neuronal migration [260]. New data also suggests that *LIS1*-deficient organoids exhibit changes in extracellular matrix properties and tissue mechanical stiffness, further exacerbating migration defects [261].

The *DCX* gene encodes a protein that stabilizes MTs and participates in their assembly [239,262,263]. Doublecortin is a neuronal MAP that plays a critical role in neuronal migration and the development of neural connections. Mutations in the *DCX* gene cause X-linked lissencephaly. In a study by Jang et al., a five-year-old girl with lissencephaly and subcortical striatal heterotopia was found to have a mutation in the *DCX* gene [264]. Another study by Tsai et al. describe a *DCX* c.785A>G, p.Asp262Gly mutation, and a functional study showed reduced ability of the mutant doublecortin to bind MTs [265].

There are several patient phenotypes of mutations in this gene: heterozygous mutations in women lead to subcortical striate heterotopia, while hemizygous mutations in men lead to lissencephaly [237,266,267]. A detailed analysis of several families with siblings affected by lissencephaly and women affected by subcortical striate heterotopia revealed a *DCX* mutation frequency of 84.6% (22 out of 26) in sporadic patients with subcortical band heterotopia (SBH) and 100% (11 out of 11) in SBH pedigrees [268]. Interestingly, unlike *Lis1*, homozygous *Dcx* gene mutations do not result in lethal outcomes, as demonstrated in mouse models [269].

The mechanism of doublecortin action is based on its ability to bind MTs and stabilize their structure, which ensures the directed migration of neurons and the growth of their processes [270,271]. As a phosphoprotein, doublecortin is a substrate for MARK1 and PKA kinases on the Ser47 residue [272]. The phosphorylation of doublecortin by these kinases reduces its binding to MTs and also determines the correct localization of doublecortin in the leading processes of migrating neurons. Another serine residue, Ser297, is phosphorylated by CDK5, which enhances the ability of doublecortin to bind to MTs *in vivo*.

It is also worth noting that doublecortin can bind to LIS1, which stabilizes MTs in the perinuclear space of the migrating neurons [273]. Phosphorylation by another kinase, MAPK8, on residues Thr321, Thr331, and Ser334, attracts doublecortin to the growth cone of the leading process.

On the other hand, binding of doublecortin to MAPK8IP1 regulates the RELN pathway, which in turn modulates actin dynamics [237].

*YWHAE* (tyrosine-3-monooxygenase/tryptophan-5-monooxygenase activation protein, epsilon) is a gene that, like *DCX*, encodes a microtubule-associated protein. Interestingly, this gene is located relatively close to *LIS1*, just 1 Mb away, on chromosome 17p. Large deletions in this region (17p13.3, which contains both *YWHAE* and *LIS1*) cause Miller–Dieker syndrome, with even more severe defects in neuronal migration compared to lissencephaly caused by the heterozygous *LIS1* mutant [237,239,274].

Patients with *YWHAE* deletions that do not affect the *LIS1* region have also shown cognitive impairments and structural brain malformations [244,275,276,277]. Analysis of five patients with deletions that affect *YWHAE* gene but do not affect *LIS1* has shown a direct correlation between genetic abnormalities and structural abnormalities in the patient brain [244]. Homozygous deletion of mouse *Ywhae* causes defects in neuronal migration.

The YWHAE protein binds to phosphorylated CDK5-NDEL1 (P-NDEL1). This interaction protects P-NDEL1 from dephosphorylation, while the functional role of phosphorylating these sites is to interact with dynein and LIS1. The formation of the P-NDEL1/YWHAE complex allows for a strong association with dynein and LIS1 along MTs. When YWHAE protein is lost, the localization of NDEL1 and LIS1 at the plus ends of MTs is disrupted [237].

*TUBA1A* (alpha subunit 1A tubulin isoform) and *TUBB2* (beta subunit 2B tubulin isoform) encode structural microtubule subunits that are highly expressed during brain development [278]. Mutations in *TUBB2* are associated with symmetric polymicrogyria and pachygyria. Pathogenic variants of *TUBA1A* have been identified in 1% of patients with classic lissencephaly and in 30% of patients with lissencephaly and cerebellar hypoplasia [279,280].

Mutations in *Tuba1a* disrupt GTP-dependent incorporation of alpha and beta tubulin into the heterodimer, leading to improper microtubule polymerization [281]. For example, the S140G mutation reduces the protein’s ability to bind GTP and form a native heterodimer, thereby preventing microtubule polymerization and neuronal migration in mice [281].

In animal models with p.Arg402Cys/p.Arg402His *Tuba1a* mutations, it was shown that MTs remain capable of integrating into the cell cytoskeleton and even assemble correctly, but they lose their ability to support the dynein motor, resulting in impaired transport of cargo. As a result, neurons retain in the VZ without reaching the CP. Later, it was shown that p.Arg402Cys/p.Arg402His substitutions specifically disrupt the interaction between MT and dynein, and the extent of dynein disruption depends on the amount of mutant tubulin expressed [282].

Mutations in the *TUBB2B* gene cause impaired neuronal migration within the IZ, while mutations in the *TUBB3* gene (an isoform of the beta subunit 3 of tubulin) alter the dynamics of MTs, disrupting the process of directed axonal growth [278,283]. *TUBG1* encodes γ-tubulin and is expressed in the developing brain of the embryo [284,285]. Two patients with *TUBG1* mutations (P367: c.1160T>C, p.Leu387Pro, P388: c.275A>G, p.Tyr92Cys) have been described, and their brain abnormalities included pachygyria/agyria with a predominance of posterior regions [284]. γ-tubulin, as a component of the microtubule nucleation center (γTuRC), is essential for the proper functioning of the microtubule cytoskeleton and neuronal migration [284].

In recent years, clinical descriptions of new *TUBA1A* variants and expanded genotype–phenotype correlations have been published [282]. These aspects explain why some tubulin mutations cause migration disorders and severe lissencephaly, while others result in milder cortical dysplasia. It seems that not only the number of MTs, but also their quality and ability to transport cargo, is important for neuronal migration [282].

The *RELN* gene encodes an extracellular matrix glycoprotein called reelin [286]. This gene is expressed by Cajal–Retzius cells, pioneer neurons that control positioning of projection neurons in the neocortex [287]. The biological effects *Reln* mutation cause changes in the properties of cell adhesion molecules in the final stages of neuronal migration [288]. The disruption of this gene causes an autosomal recessive form of lissencephaly, along with other pathologies (such as cerebellar hypoplasia, hippocampal malformations, etc.) in humans.

A link has been shown between moderate lissencephaly and a homozygous mutation in the *RELN* splicing region. A prenatal case of lissencephaly with a c.2972G>A mutation in the *RELN* gene has been described [289]. Mutations in the *VLDLR* gene (very-low-density lipoprotein receptor) also lead to impaired cortical architecture similar to *RELN* mutation, but in a more moderate form [290]. Mice carrying a mutation in this gene showed a lack of a distinct marginal zone layer and inverted “upside down” cortical architecture [237,291].

As an extracellular protein, RELN binds to VLDLR, LRP8 (low-density lipoprotein receptor 8) and ApoER2 (apolipoprotein E receptor 2) receptors, and activates downstream effector DAB1 [292,293,294].

After RELN receptor binding t—SRC, FYN kinases are activated. The complex effect is the transmission of the signal to the intracellular PI3K-AKT-GSK3beta pathway, which in turn increases the phosphorylation of MAPT (microtubule-associated protein tau) and MTAP1B by CDK5 kinase [294,295,296]. This phosphorylation has a direct effect on the dynamics and stability of MTs [237]. The same activation of receptors by this signaling molecule leads to an increase in the enzymatic activity of another kinase, LIMK1 (LIM-domain-containing protein kinase 1), which phosphorylates cofilin. Phosphorylation of cofilin in turn leads to its inactivation and, as a result, increases the stability of actin stress fibers [237,297,298,299].

There are two genes that have been shown to be associated with lissencephaly, and they directly encode structural elements of actin. The *ACTB* (actin beta) gene encodes one of the six structural proteins of actin, β-actin. The product of the *ACTG1* (actin gamma 1) gene is also a structural non-muscle actin, γ-actin. Mutations in these genes are linked with BRWS (MIM#243310), with lissencephaly as a part of the phenotype.

Whole-exome sequencing in three probands found mutations of cytoplasmic actin *ACTB* and *ACTG1* in one and two probands, respectively, and sequencing of both genes in fifteen additional patients revealed pathogenic mutations in all probands, including two recurrent de novo mutations (*ACTB* p.Arg196His and *ACTG1* p.Ser155Phe) [223]. Cases with deletions of the region containing the *ACTB* gene and mutations in the coding region of the gene have been described, namely c.1097dupG; p.Ser368LeufsTer13, c.1117A>T; p.Lys373Ter, c.329delT, which also confirmed the unambiguous link between the loss of *ACTB* gene function and the pleiotropic clinical syndrome [225]. *ACTB* and *ACTG1* mutations (p.Arg196His and p.Ser155Phe) cause increased F-actin level in a cell and multiple abnormal F-actin-rich filopodia, resulting in increased cell perimeter [223]. The disruption of F-actin polymerization dynamics and altered ATP hydrolysis rate altered the assembly of AFs, leading to impaired neuronal migration [223,300].

### 4.3. Corpus Callosum Dysgenesis

The corpus callosum is the largest interhemispheric commissure in mammals, a massive bundle of white matter in the brain that consists of almost 200 million axons in humans [301,302]. Agenesis of the corpus callosum is a congenital malformation in which this interhemispheric tract is completely or partially absent [303]. This condition is one of the most common cortical malformations, with an incidence rate of 1.8 to 2.5 per 10,000 live births [304].

It can occur both in isolation and as part of numerous congenital syndromes [305]. Despite the clinical significance and relatively high prevalence of corpus callosum agenesis, the neurocognitive profile of patients is characterized by significant heterogeneity and a wide range of functional outcomes [303,306,307].

The genetics of corpus callosum agenesis are multifaceted, but genes that regulate the neuronal cytoskeleton play an important role [2,308].

It has been shown that destabilization of the cytoskeleton prevents normal navigation and elongation of commissural axons across the midline of the brain [309]. Disruption of microtubule dynamics leads to collapse of the growth cone [309,310,311,312], which is a central factor in the pathogenesis of dysgenesis and agenesis of the corpus callosum. As a result of the collapse, axons lose their ability to perceive signals from guidance molecules and are unable to cross the midline [313,314]. This leads to their premature termination or aberrant re-routing, resulting in the formation of Probst bundles instead of a fully developed corpus callosum [315].

Genes encoding microtubule structural proteins (tubulins) play a fundamental role in the pathogenesis of corpus callosum agenesis [316,317]. All mutations reported in the *TUBA1A*, *TUBB2B*, *TUBB3*, and *TUBB* genes are heterozygous missense variants [318,319]. Pânzaru et al. [320] suggest that this may indicate a dominant-negative effect.

Unlike mutations that lead to complete loss of protein, missense substitutions cause the synthesis of abnormal tubulin, which is incorporated into MTs and disrupts their dynamic stability. This results in defects in neuronal migration and severe impairments in axonal navigation.

Missense variants of the *TUBA1A* gene (*LIS3*, *TUBA3*, *B-ALPHA-1*) associated with commissural dysgenesis are the most common in clinical practice [317,318,319,321]. A large-scale meta-analysis of clinical cases conducted by Hebebrand et al. [319] covered data from 166 patients and showed that pathogenic substitutions tend to cluster in the C-terminal domain of the protein, particularly in the highly conserved position of Arg402.

Similar conclusions about the structural localization of mutations were confirmed in an extended review published in 2023 [322]. In the study by Buscaglia et al. [323], they examined the *Tuba1a*^ND/+^ mouse line, which was identified using ENU mutagenesis. The authors demonstrated that reduced expression of *Tuba1a* inhibited neurite growth compared to the control group. This defect prevented axons from crossing the midline, making it impossible to form the corpus callosum.

The *TUBB2B* gene (CDCBM7, PMGYSA, bA506K6.1) encodes a neurospecific β-tubulin isoform that plays a key role in the early development of the CNS [324]. Studies in mice have confirmed its intense expression in neuronal precursor cells and migrating neurons [325,326,327]. Additionally, it has been shown to be preferentially localized in MTs in the peripheral region of the growth cone [326]. The spatial and temporal expression patterns of *Tubb2b* correlate with defects in the directed growth of axons, which have been observed both in experimental mouse models [318,328] and in clinical cases of patients with mutations in this gene associated with corpus callosum agenesis [329,330,331,332,333].

In addition to the above-mentioned factors, mutations in the *TUBB3* (*CDCBM, CFEOM3, FEOM3, TUBB4*) and *TUBB2A* (*CDCBM5, TUBB, TUBB2*) genes significantly contribute to the development of corpus callosum pathologies, as shown in [334,335,336,337] and [329,338,339,340,341,342], respectively.

One of the genes associated with corpus callosum agenesis is *CDK5RAP2* (*C48*, *Cep215*, *MCPH3*). Its protein product, the regulatory subunit 2 of cyclin-dependent kinase 5, is a pericentriolar structural protein that coordinates the functions of MTs through its interaction with the γ-TuRC [320,343]. This gene is predominantly expressed in the structures of the corpus callosum, indicating its key role in the morphogenesis of this commissure [344]. Jouan et al. [345] identified compound heterozygous missense variants in the *CDK5RAP2* gene with isolated corpus callosum agenesis.

It is assumed that mutations in the *CDK5RAP2* gene with residual protein function may lead to isolated corpus callosum agenesis. However, variants with complete loss of function cause a more severe phenotype, autosomal recessive primary microcephaly, in which a general decrease in brain volume is combined with partial or complete agenesis of the commissure [345].

The DPYSL family of cytosolic phosphoproteins plays a key role in the development of the nervous system. They coordinate the dynamics of the cytoskeleton through direct interactions with tubulin and actin [346].

Pathogenic variants of the *DPYSL2* (*CRMP-2*) and *DPYSL5* (*CRMP-5*) genes have been associated with abnormalities of the corpus callosum (hypoplasia, agenesis), as described in detail in the review by Desprez et al. [346]. Of particular interest is the competitive interaction between these proteins, which regulates the growth of dendrites [347,348]. While phosphorylation of DPYSL2 inhibits growth by weakening its association with tubulin, phosphorylation of DPYSL5 (via GSK3β) blocks tubulin polymerization.

This fine-tuning of early neuronal development through axon elongation and dendritic growth is critical for the formation of the corpus callosum and normal cognitive development [349,350]. In their work, Desprez et al. [349] expand on this understanding by describing the contribution of heterozygous de novo variants of the *DPYSL5* gene in five probands with corpus callosum agenesis. Thus, the disruption of the delicate balance between *DPYSL2* and *DPYSL5* activity plays a central role in the pathogenesis of corpus callosum agenesis, and the identification of such genotype–phenotype correlations highlights the clinical significance of genetic screening for DPYSL-associated conditions in the diagnosis of corpus callosum malformations.

WDR47 protein (Nemitin) is critical for stabilizing MTs in the growth cone and maintaining mitochondrial homeostasis, which ensures the survival of corpus callosum neurons [351,352,353,354]. Studies in mouse models show that the complete loss of *Wdr47* leads to the loss of the corpus callosum and neonatal death, while partial loss of function causes its thinning due to defects in axon elongation. Clinical observations in five unrelated families with biallelic *WDR47* variants revealed corpus callosum dysgenesis in combination with microcephaly and enlarged ventricles [352]. These results suggest that *WDR47* is responsible for the structural integrity of the corpus callosum and the survival of neurons during development.

The product of the *ACTG1* gene, γ-actin, is a ubiquitously expressed cytoskeletal protein [226,355,356]. Its pathogenic variants underlie Baraitser–Winter type 2 syndrome [357], characterized by facial dysmorphism, cortical abnormalities (pachygyria, heterotopia), microcephaly, and severe neurological and cognitive impairments [358]. Along with the classic features, a number of patients with this syndrome have been reported to have agenesis of the corpus callosum [359]. It has been shown that pathogenic variants of the *ACTG1* gene are associated with abnormal expression of the SNAP-25 protein, which is critical for axon elongation and the spatial organization of the corpus callosum [359,360].

The protein product of the *RAC3* gene is a regulator of the actin component of the cytoskeleton and plays a crucial role neurogenesis [361,362,363]. RAC3 protein is essential for the growth of neurites, the formation of axons and dendrites, synaptogenesis, and neuronal migration [363,364,365,366,367,368]. Pathogenic variants of *RAC3* are associated with corpus callosum agenesis, colpocephaly, and small brain volume [363,369,370,371,372,373].

Missense mutations in *SIP1* p.Tyr1055Cys, p.Ser1071Pro, and p.His1045Arg in the highly conserved zinc finger domain (C-ZF) have been described in patients with the phenotype of Mowat–Wilson syndrome. This gene contributes to the development of the neural tube, regulates the differentiation and migration of neural crest cells, and modulates GABAergic transmission [374]. It has been established that the deletion of *Sip1* in *Sip1* fl/fl Nex Cre/w mutant mice resulted in the absence of the corpus callosum and anterior commissure, which were replaced by Probst fibers [44].

Another protein, ninein, which was implicated in corpus callosum genesis, was identified in a mouse model of the Mowat–Wilson syndrome *Nin* gene [44]. The protein localizes to the somatodendritic compartment of neurons and binds the minus end of microtubules [375].

Ninein, as a protein that interacts with the minus end of microtubules, affects the stability and growth of microtubules, as well as the dynamics of the plus end [44]. It is also necessary for the stabilization of centrosomal microtubules. It has been shown that the expression of ninein is also required for the formation of apico-basal microtubules [376].

### 4.4. Synaptopathies

Synaptic communication between nerve cells is the key to the nervous system’s functioning. Various defects in either the structure or function of synaptic contacts can lead to a wide range of pathologies collectively referred to as synaptopathies.

This category of diseases includes intellectual disability, autism spectrum disorders (ASDs), attention deficit hyperactivity disorder (ADHD), and epileptic encephalopathies [377]. According to the WHO, ASD affects up to 0.76% of children worldwide [378]. As for intellectual disability, the prevalence rate was 1.74% in 2019 [379]. Recently, there has been an increasing body of evidence linking mutations in cytoskeletal genes to the development of synaptopathies.

Pathogenic variants in proteins that regulate the activity of Rho GTPase family members are common causes of various neurodevelopmental disorders [361]. For example, oligophrenin-1 (*OPHN1*) was one of the first Rho-associated genes for intellectual disability, and subsequent studies have also confirmed its role in the development of schizophrenia, epilepsy, and ASD [380,381]. This protein belongs to the GAP family and acts as a negative regulator for RhoA and Rac1 [382]. *Ophn1* is widely expressed in the developing and adult nervous system, with a predominance in the cerebral cortex and hippocampus.

In neurons, the protein was found not only in axons and dendrites, but also on both sides of synapses, where it regulates the maturation of dendritic spines and the recycling of SVs [381,383]. Cortical neurons derived from iPSCs from fibroblasts of two patients with *OPHN1* mutations exhibited an immature morphological phenotype [384].

*In vivo* and *in vitro* experiments have shown that the deficiency in this gene leads to a significant decrease in the density of mature dendritic spines [383], disruption of SV endocytosis and AMPAR internalization [385], an increase in the number of excitatory synapses, loss of LTD [381], and a decrease in evoked and spontaneous excitatory and inhibitory postsynaptic current (EPSCs and IPSCs) [386,387].

In addition, *Ophn1* knockout mice exhibited defects in spatial memory, social behavior, lateralization, and hyperactivity [383]. These animals also showed increased RhoA GTPase activity [385]. Taken together, these findings suggest that OPHN1 plays a crucial role in organizing the synaptic cytoskeleton by regulating members of the Rho family of small GTPases, which govern the interaction between MT and actin in the developing nervous system [385,388].

MAP1B is a member of the MAP family that plays a critical role in axon growth, dendritic spine maturation, and synapse formation by regulating the dynamics of the actin and microtubule cytoskeletons through its interaction with the guanosine nucleotide exchange factors GEF-H1 and Tiam, which regulate the activity of the small GTPases RhoA and Rac1 [389]. This protein is highly expressed in early stages of neural development in neurons and glial cells, as well as in highly plastic regions of the adult brain, such as the olfactory bulbs, hippocampus, cortex, and cerebellum [390,391].

Clinical studies have shown that variants of the *MAP1B* gene are associated with intellectual disability, ADHD, ASD, and epilepsy [391,392,393].

It was found that *Map1b* deficiency leads to delayed growth and reduced axon elongation rate [394], impaired LTD, defective LTD-induced AMPAR endocytosis in dendritic spines [389], reduced amplitude of miniature excitatory postsynaptic currents (mEPSCs) [395], decreased synapse number, increased proportion of orphan presynaptic terminals (OPTs), and reduced density of SV and large dense core vesicles [396].

In addition, changes in the cytoskeleton were observed at the subcellular level, manifested in the disruption of microtubule dynamics in axons [394] and actin in dendritic spines [389]. This dysregulation may be driven by a decrease in Rac1 activity and an increase in RhoA activity [395]. The decrease in Rac1 activity may be attributed to a reduction in the accumulation of Tiam1 in the synaptic compartment [389]. These findings further support the critical role of Rho GTPase regulators in determining synaptic function through their influence on cytoskeletal dynamics.

Another member of the MAP family that plays an important role in the functioning of synaptic structures is MAP6 or STOP. Mammals have several isoforms encoded by the single gene *MAP6*. In neurons, the N-STOP and E-STOP isoforms are primarily expressed, where they participate in the stabilization of MTs, making them resistant to cold and nocodazole [397]. Additionally, this protein has been shown to play a role in the formation and maintenance of dendritic spines by stabilizing actin and enhancing its nucleation in response to neuronal activation, a key event in synaptic plasticity [398]. Mutations in *MAP6* have been linked to the development of ASD [399] and schizophrenia [400] in humans.

Mice with a knockout of this gene exhibit various cognitive and behavioral abnormalities, including social isolation, impaired social learning and sensory processing [400], disorganized activity, impaired parenting, and anxiety [401]. These behavioral phenotypes may be attributed to multiple synaptic dysfunctions, as supported by several experimental studies.

In the hippocampus of *Map6*-deficient mice, SV pool was depleted, causing defects in short-term and long-term plasticity [401], a decrease in the density of dendritic spines [398], and a reduction in the mRNA levels of synaptic proteins (synaptofusin, GAP-43, VGlut1, and spinophilin) [402]. In addition, there were abnormalities at the cytoskeletal level, which manifested as reduced cold-resistance of MTs [401] and defects in the stabilization of AFs [398].

In a cohort study of individuals with intellectual disability, five patients were identified that carried homozygous frameshift variants in the *FMN2* gene [403]. The product of this gene is a member of the formin protein family, which is involved in regulating the dynamics of both actin and MTs, thereby participating in the nucleation of AFs [404], generating adhesion forces by filopodia, and stabilizing the growth cone during the directed elongation of neurites [3,405].

iPSC-derived neurons with *FMN2* deficiency had a 43% reduction in synaptic density compared to healthy control neurons. Similar abnormalities were also observed in *Fmn2*-deficient mice, which had a 32% reduction in the density of dendritic spines in the granular neurons of the dentate gyrus of the hippocampus [403].

Another important protein involved in synaptogenesis is cortactin-binding protein 2 (CTTNBP2), which is named due to its ability to interact with cortactin, a ubiquitously expressed regulator of the actin cytoskeleton that controls the polymerization of F-actin and the branching of AFs by interacting with the Arp2/3 complex [406]. In addition to regulating the dynamics of F-actin, this protein also plays a role in determining the stability of MTs.

In clinical studies, *CTTNBP2* was identified as a gene associated with ASD [407]. When this was modeled in *Cttnbp2* knockout mice, social behavior, spatial memory, learning, and vocal communication were impaired [408]. These behavioral patterns may be explained by alterations in the structure and function of synapses, such as reduced density, width, and length of dendritic spines, decreased length and thickness of the postsynaptic density, reduced pool of presynaptic vesicles, and decreased frequency of mEPSCs [407].

Thus, this chapter summarizes data on pathogenic variants of key genes of various cytoskeleton components and their regulators in the context of major NDDs (Table 1). The genes included in Table 1 are characterized not only by the presence of clinical studies that have established genotype–phenotype correlations, but also by the existence of experimental models that have allowed us to study the pathogenesis of these conditions. Taken together, these findings highlight the critical role of MTs, AFs, and IFs in neurodevelopmental processes. However, we still do not have a comprehensive and accurate understanding of the molecular mechanisms underlying the development of NDDs, which points out the need for further research.

## 5. Conclusions

This review summarizes current data on the role of all three main components of the cytoskeleton, namely microtubules, actin filaments, and intermediate filaments, in the context of cortical development and the pathogenesis of neurodevelopmental disorders. A comprehensive analysis of the literature demonstrates that the neuronal cytoskeleton functions as a unified, highly dynamic system, where each component plays a unique and indispensable role.

While microtubules are critical for proliferation, polarization, and intracellular transport, and actin filaments are essential for directed migration and synaptic plasticity, intermediate filaments, previously considered primarily as passive structural elements, provide mechanical integrity to neurites and modulate synaptic function.

The collected data convincingly demonstrate that pathogenic variants of genes encoding proteins of various cytoskeleton components underlie a wide range of NDDs, from classic tubulopathies associated with cortical malformations to disorders associated with mutations in actin filament genes. Intermediate filaments, in turn, were previously underestimated as factors of neuropathologies and have long been viewed solely as passive structural elements. However, the current research demonstrates that IFs are active participants in maintaining the mechanical integrity of neurites and neuronal somas, as well as synaptic plasticity. The contribution of pathogenic variants of IF-encoding genes to the mechanisms of various NDDs remains unexplored. Available data are fragmentary and primarily based on in vitro studies, highlighting the importance of developing research in this area.

Of particular importance is the fact that even minor defects in the regulation of the cytoskeleton in the early stages of ontogenesis can cause severe disorders that manifest themselves in the form of cognitive and behavioral abnormalities in the postnatal period. A comprehensive approach is required in future research to investigate the contribution of the cytoskeleton to the pathogenesis of NDD.

First of all, there is a need to understand cross-interactions. Although the roles of MTs, AFs, and IFs in individual aspects of neurogenesis have been well-studied, their coordinated interactions remain poorly understood. Therefore, a critical area of research is the study of the molecular mechanisms that coordinate the functioning of all three components of the cytoskeleton. The following remains largely unknown: How do signals from the extracellular microenvironment simultaneously regulate the dynamics of microtubules and actin in the growth cone? How are intermediate filaments integrated into these processes? Understanding these cross-interactions will reveal common pathogenic bases that are characteristic of different forms of NDDs.

Secondly, improving and expanding experimental models of cytoskeletal component dysfunction by inactivating the genes encoding them are essential. The need to develop high-quality *in vitro* models stems from the limitations of traditionally used primary neuronal cultures, which do not reproduce the three-dimensional architecture of the brain. For this purpose, the use of cerebral organoids based on induced pluripotent stem cells from patients with pathogenic variants in cytoskeletal genes appears promising. The use of such organoids will allow modeling key stages of corticogenesis, followed by a detailed assessment of the impact of cytoskeletal dysfunction on each of them. Improving *in vivo* models, in turn, is necessary to establish a causal relationship between cytoskeletal dysfunction and the characteristic NDD phenotype, particularly for those cytoskeletal genes for which such a relationship has not previously been demonstrated. Utilizing electrophysiology tools and behavioral tests will allow better understanding of correlations between cytoskeletal defects and cognitive–behavioral dysfunctions in patients.

Thirdly, from a translational research perspective, a key challenge is improving diagnostics. Integrating knowledge about genes encoding cytoskeleton proteins into modern diagnostic panels based on next-generation sequencing methods will enhance the detection of genetic causes of NDDs. This is particularly important for patients with undifferentiated forms of developmental delay, where cytoskeleton pathology may be a crucial but not an obvious factor. Thus, the comprehensive analysis of the cytoskeleton presented in this review not only deepens our fundamental understanding of neurogenesis, but also provides a solid foundation for future research aimed at early diagnosis of a wide range of neurodevelopmental disorders.

## Figures and Tables

**Table 1:** Key cytoskeleton genes and associated NDDs.

NDD	The Stage of Corticogenesis	The Cytoskeleton Element	Genes [Reference]
Microcephaly	Proliferation	MTs	*PRUNE1* [204] *NIN* [44,374,376]
		AFs	*MTTS2* [221] *ACTB* [228] *ACTG1* [228]
		IFs	-
	Apoptosis	MTs	*TUBB5* [197,199] *SPOUT1* [207]
		AFs	-
		IFs	-
Lissencephaly	Migration	MTs	*LIS1* [237,253,255,256,257,259,260,261] *DCX* [237,272,273] *YWHAE* [409] *TUB1A1* [281,282] *TUBB2* [278] *TUBB3* [283] *TUBG1* [284] *RELN* and *VLDLR* [237,297,298,299]
		AFs	*LIS1* [237,253,255,256,257,259,260,261] *DCX* [237,272,273] *RELN* and *VLDLR* [237,297,298,299] *ACTG1* [223] *ACTB* [223,300]
		IFs	-
Corpus callosum dysgenesis	Axonogenesis	MTs	*TUBA1A* [320,323] *TUBB2B* [318,320,328] *TUBB3* [320,335] *TUBB2A* [320] *CDK5RAP2* [345] *DPYSL2* [347,350] *DPYSL5* [347,350] *WDR47* [351,353,354] *NIN* [44,374,376]
		AFs	*ACTG1* [359,360] *RAC3* [363,364,365,366]
		IFs	-
Synaptopathies	Synaptogenesis	MTs	*OPHN1* [381,383,384,385,386,387] *MAP1B* [389,394,395,396] *MAP6* [398,400,401,402] *FMN2* [403] *CTTNBP2* [407]
		AFs	*OPHN1* [379,381,382,383,384,385] *MAP1B* [387,392,393,394] *MAP6* [396,398,399,400] *FMN2* [401] *CTTNBP2* [405]
		IFs	-

## Data Availability

No new data were created or analyzed in this study. Data sharing is not applicable to this article.

## Abbreviations

The following abbreviations are used in this manuscript:

ADHD	Attention deficit hyperactivity disorder
AF	Actin filaments
AMPAR	α-amino-3-hydroxy-5-methyl-4-isoxazolepropionic acid receptor
aRGC	Apical radial glial cells
ASD	Autism spectrum disorders
BRWS	Baraitser–Winter syndrome
CNS	Central nervous system
CP	Cortical plate
ENU	N-ethyl-N-nitrosourea
EPSC	Excitatory Postsynaptic Current
ER	Endoplasmic reticulum
GABA	γ-aminobutyric acid
GAP	GTPase-activating proteins
GFAP	Glial fibrillary acidic protein
GMPCPP	Guanidyl 5′-α,β-methylenediphosphonate
GRIP	γ-tubulin ring proteins
IF	Intermediate filaments
IPSC	Inhibitory Postsynaptic Current
IPSCs	Induced pluripotent stem cells
IZ	Intermediate zone
LIMK	LIM kinase
LTD	Long-term depression
LTP	Long-term potentiation
MAPs	Microtubule-associated proteins
mEPSC	Miniature excitatory postsynaptic currents
MT	Microtubules
MTOC	Microtubule organizing center
NDDs	Neurodevelopmental disorders
NF-H	Heavy neurofilament
NF-L	Light neurofilament
NF-M	Medium neurofilament
NMDAR	N-methyl-D-aspartate receptor
SBH	Subcortical band heterotopia
SV	Synaptic vesicles
VZ	Ventricular zone
WASP	Wiskott–Aldrich syndrome protein
γ-TuRC	γ-tubulin ring complex
